# Bilateral Segmental Testicular Infarction

**DOI:** 10.1100/tsw.2007.146

**Published:** 2007-02-19

**Authors:** Aaron Bayne, Brad Koslin, Siamak Daneshmand

**Affiliations:** ^1^ Division of Urology and Renal Transplantation, Oregon Health and Science University, Portland, OR, USA; ^2^ Department of Diagnostic Radiology, Oregon Health and Science University, Portland, USA

**Keywords:** testicle, infarction, ultrasound, scrotal, emergency, segmental, ischemia

## Abstract

Segmental testicular infarction is a rare entity with fewer than 40 cases documented in the literature. It frequently mimics an acute scrotum presenting with pain and swelling. Difficulty distinguishing benign from malignant lesions on imaging has led to radical orchiectomy in the past. With improvements in imaging, this condition may be treated more conservatively. We present the first case of bilateral segmental testicular infarction and discuss management options.

## CASE PRESENTATION

A 31-year-old healthy male physician presented to the Emergency Room with 3 h of acute onset right scrotal pain. At arrival, the pain had spontaneously subsided. Scrotal exam was normal and the patient denied any history of trauma. Color Doppler ultrasound revealed a region of central hypovascularity on the right and a normal left testis (Fig. 1). The patient was discharged home with planned elective orchiopexy for presumed spontaneous detorsion of his testicle. Less than 24 hours later, he represented to the ER with acute pain and swelling of his left testis. The patient had normal tumor markers (AFP, bHCG, LDH), normal urinalysis, and normal serum chemistries. He underwent a repeat scrotal ultrasound that revealed a rapid change in the appearance of his right testicle consistent with an evolving infarction and a new focal lesion in the left testis with absent blood flow on color Doppler (Fig. 2). There were no areas of hyperemia to suggest a recent detorsion, nevertheless, the patient underwent bilateral orchiopexy to rule out this possibility. At the time of surgery, the right testicle appeared normal. The left testicle appeared edematous with a mild reactive hydrocele. The spermatic cord was not torsed and there were no palpable masses in either testicle. Rheumatologic and serologic evaluation failed to reveal any cause. One month later, repeat scrotal ultrasound showed decrease in size of both testicular lesions, the left showing heterogeneous decreased echogenicity, and no new areas of hypovascularity in either testicle consistent with evolving infarction (Fig. 3).

**A**

**B**

**FIGURE 1**. (A) Note the area of hypovascularity in the right testis; (B) normal left testis.

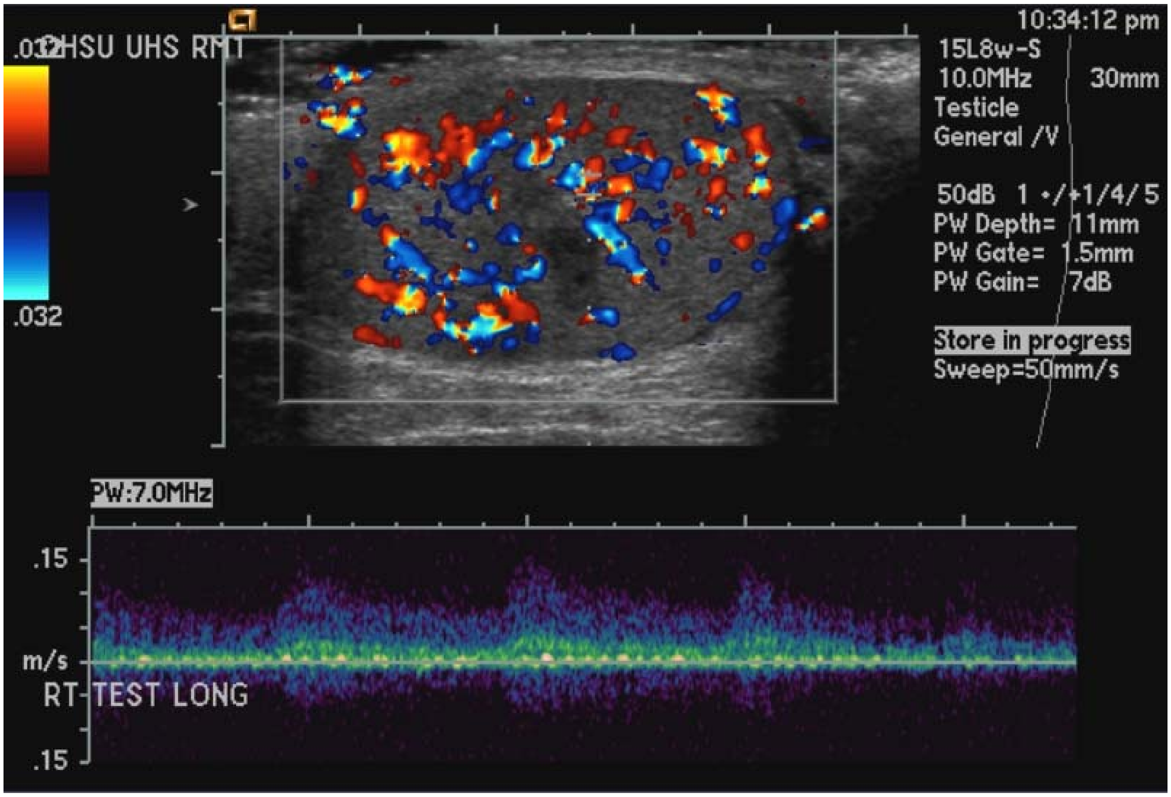

A

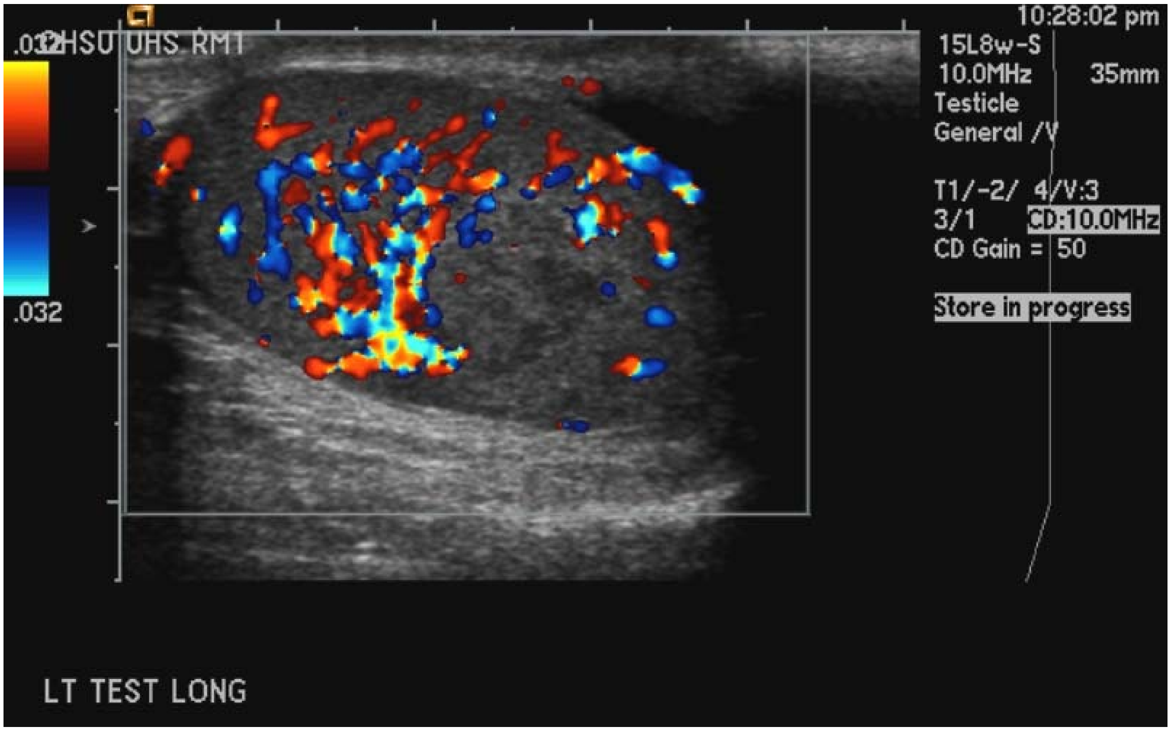

B

**FIGURE 2.** (A) Rapid evolution of right testis lesion; (B) new focal lesion in left testis.

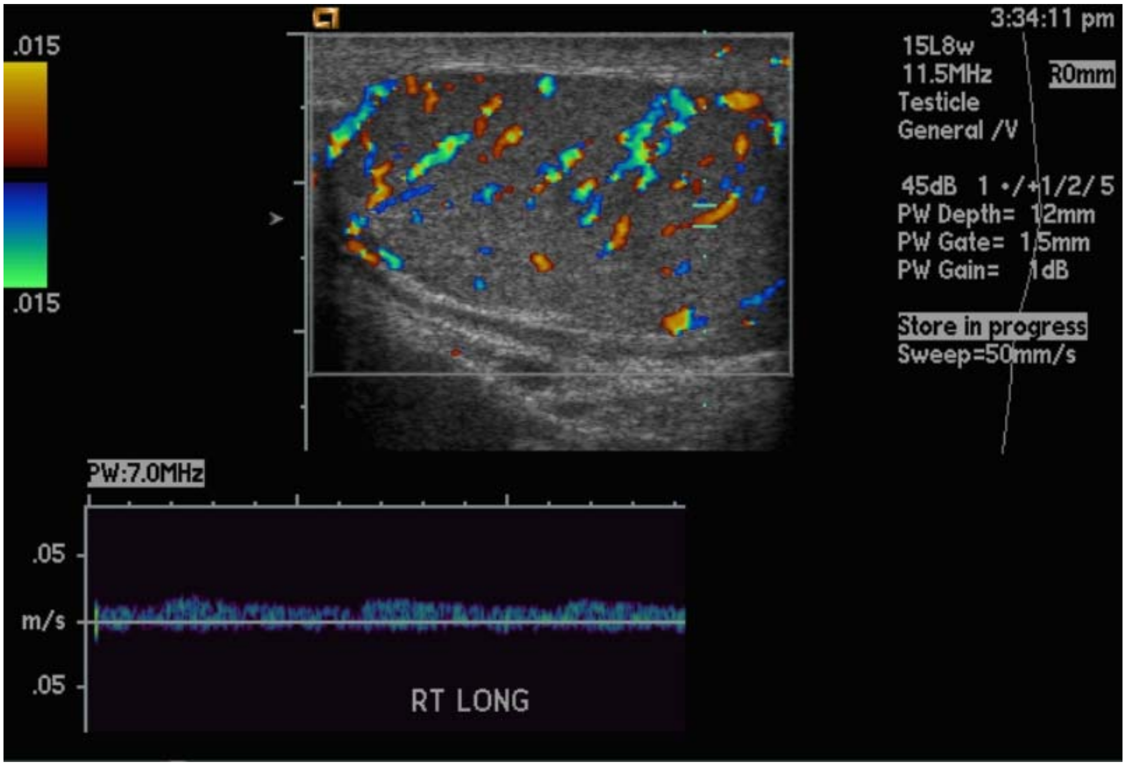

A

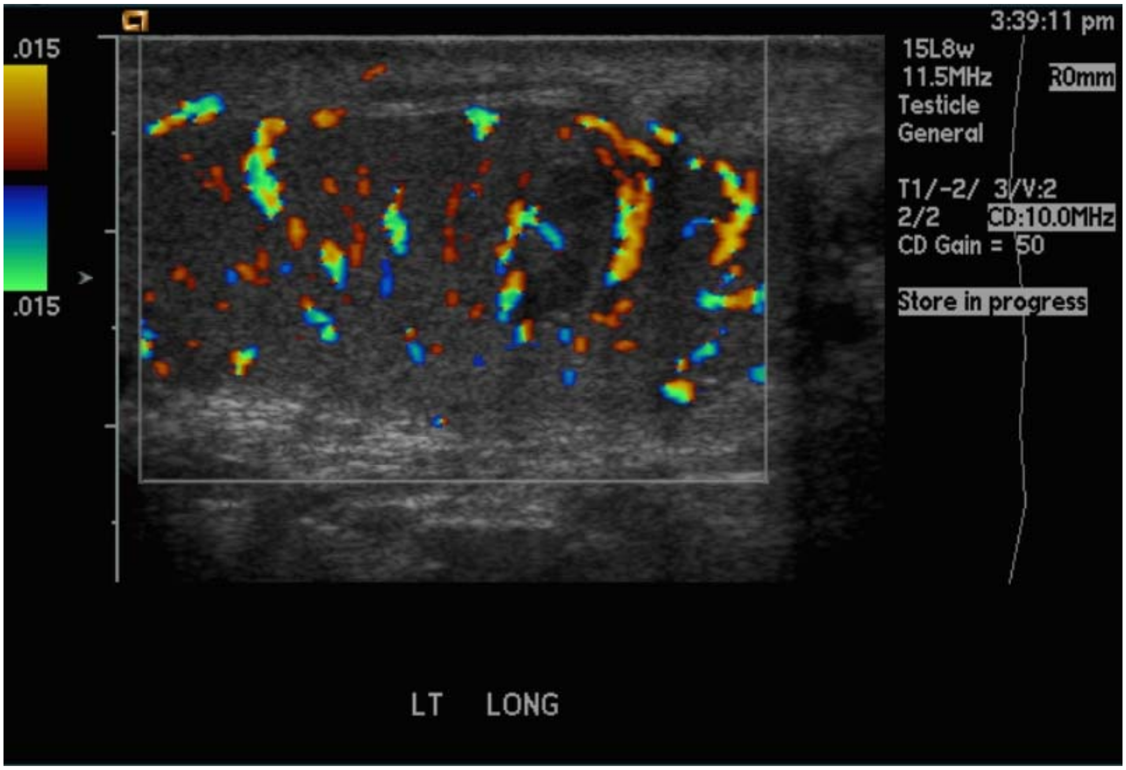

B

**FIGURE 3**. Improvement in (A) right testicle and (B) left testicle appearance and blood flow.

## DISCUSSION

Segmental testicular infarction is a rare entity and is usually idiopathic. With improved imaging using color flow Doppler and MRI aiding in diagnosis, more conservative management strategies have evolved[1]. Recently, authors have advocated testis-sparing surgery[2] and other observations along with follow-up imaging[3]. We report the first case of bilateral segmental testicular infarction.

Consecutive day ultrasound allowed for observation of the *de novo* occurrence of the left testis lesion and the changing appearance of the right lesion. Our differential diagnosis included testicular tumor, epididymoorchitis, vasculitis, and systemic malignancy. With negative tumor markers and close ultrasound follow-up, we were able to safely spare both testis and document improvement of both lesions at 1 month.

## CONCLUSION

In patients with segmental testicular infarction, differentiating between a benign lesion and a neoplasm can be difficult. With appropriate imaging, negative tumor markers, close follow-up, and careful patient selection, surgery can be avoided. In cases where the scrotal ultrasound suggests a focal segmental infarction, we suggest a repeat ultrasound be performed within 24 h to rule out the possibility of tumor.
